# Preliminary Assessment of Reference Region Quantification and Reduced Scanning Times for [^18^F]SynVesT-1 PET in Parkinson's Disease

**DOI:** 10.1155/2023/1855985

**Published:** 2023-08-11

**Authors:** Kelly Smart, Carme Uribe, Kimberly L. Desmond, Sarah L. Martin, Neil Vasdev, Antonio P. Strafella

**Affiliations:** ^1^Brain Health Imaging Centre, Centre for Addiction and Mental Health, 250 College St., Toronto, ON, Canada M5T 1R8; ^2^Department of Psychiatry, University of Toronto, 250 College St., Toronto, ON, Canada M5T 1R8; ^3^Unitat de Psicologia Medica, Departament de Medicina, Institute of Neuroscience, Universitat de Barcelona, Barcelona, Spain; ^4^Edmond J. Safra Parkinson Disease Program, Neurology Division, Toronto Western Hospital & Krembil Brain Institute, University Health Network, University of Toronto, 399 Bathurst Street, Toronto, ON, Canada M5T 2S8

## Abstract

Synaptic density in the central nervous system can be measured *in vivo* using PET with [^18^F]SynVesT-1. While [^18^F]SynVesT-1 has been proven to be a powerful radiopharmaceutical for PET imaging of neurodegenerative disorders such as Parkinson's disease (PD), its currently validated acquisition and quantification protocols are invasive and technically challenging in these populations due to the arterial sampling and relatively long scanning times. The objectives of this work were to evaluate a noninvasive (reference tissue) quantification method for [^18^F]SynVesT-1 in PD patients and to determine the minimum scan time necessary for accurate quantification. [^18^F]SynVesT-1 PET scans were acquired in 5 patients with PD and 3 healthy control subjects for 120 min with arterial blood sampling. Quantification was performed using the one-tissue compartment model (1TCM) with arterial input function, as well as with the simplified reference tissue model (SRTM) to estimate binding potential (BP_ND_) using centrum semiovale (CS) as a reference region. The SRTM2 method was used with *k*_2_′ fixed to either a sample average value (0.037 min^−1^) or a value estimated first through coupled fitting across regions for each participant. Direct SRTM estimation and the Logan reference region graphical method were also evaluated. There were no significant group differences in CS volume, radiotracer uptake, or efflux (*ps* > 0.47). Each fitting method produced BP_ND_ estimates in close agreement with those derived from the 1TCM (subject *R*^2^s > 0.98, bias < 10%), with no difference in bias between the control and PD groups. With SRTM2, BP_ND_ estimates from truncated scan data as short as 80 min produced values in excellent agreement with the data from the full 120 min scans (bias < 6%). While these are preliminary results from a small sample of patients with PD (*n* = 5), this work suggests that accurate synaptic density quantification may be performed without blood sampling and with scan time under 90 minutes. If further validated, these simplified procedures for [^18^F]SynVesT-1 PET quantification can facilitate its application as a clinical research imaging technology and allow for larger study samples and include a broader scope of patients including those with neurodegenerative diseases.

## 1. Introduction

Positron emission tomography (PET) with radiotracers selective for synaptic glycoprotein 2A (SV2A), such as [^11^C]UCB-J and [^18^F]SynVesT-1, represents a cutting-edge method to measure synaptic density *in vivo* in the living brain. Molecular imaging of SV2A with PET has been used to identify alterations in the brain's molecular architecture in a range of neuropsychiatric disorders. This method is of particular interest in neurodegenerative disorders such as Parkinson's disease (PD) and Alzheimer's disease [1–3], where measures of synaptic change can offer a uniquely specific and highly sensitive method to detect and track loss of synaptic connections in specific brain regions. Impairment in synaptic function or plasticity may be a fundamental pathological process beginning well before clinical symptoms emerge [1]; hence, relative to established neuroimaging measures, SV2A PET could offer earlier diagnosis or complementary information for understanding disease processes. PD is a progressive neurodegenerative disorder characterized by loss of dopaminergic terminals in the substantia nigra along with broader neural deficits [4, 5]. A number of neuroimaging strategies using PET or magnetic resonance imaging (MRI) have advanced our understanding of disease course by tracking changes in measures such as glucose metabolism, protein aggregates, or cortical thickness [6], but information is only beginning to be available at the level of the synapse.

Clinical PET studies of PD using the SV2A radiotracer [^11^C]UCB-J have identified reductions in synaptic density in substantia nigra as well as other midbrain nuclei and cortical regions [7–9]. Similar to [^11^C]UCB-J, [^18^F]SynVesT-1 quantification can be performed using the one-tissue compartment model (1TCM) fitted to brain data and an arterial input function derived from blood sampling across the duration of the PET scan [10–12]. This typically requires catheterization of the radial artery, a technically demanding and physically or psychologically uncomfortable procedure for participants. Placing and maintaining an arterial catheter for the duration of a two-hour scan can be particularly challenging in elderly participants and those with movement disorders like PD. A noninvasive method to quantify SV2A binding in these patients and a shorter total scan time would facilitate recruitment and successful scanning in studies of people with PD.

Noninvasive quantification of PET signals can be achieved using a reference region which does not express the binding target and can therefore provide a proxy estimate of nondisplaceable (i.e., free and nonspecifically bound) radiotracer in brain. For SV2A PET, the white matter centrum semiovale (CS) region has been thoroughly investigated to generate estimates of binding potential (BP_ND_) of [^11^C]UCB-J and [^18^F]SynVesT-1 [13–15]. While a small amount of specific SV2A binding is observed in this region, BP_ND_ values using reference region quantification typically show excellent agreement with values obtained from arterial blood sampling, and assuming no group differences in reference region uptake, bias is likely to be minimal. However, this protocol must be validated in each study population, and disease-related differences may be a particular concern for neurodegenerative disorders where disease processes could theoretically affect the integrity of the white matter reference region. To date, preliminary work has supported the suitability of reference region modeling of [^18^F]SynVesT-1 using the simplified reference tissue model (SRTM) in patients with Alzheimer's disease [13], but these methods have not yet been examined in PD. The goal of this work was to validate reference region modeling methods for [^18^F]SynVesT-1 quantification in patients with PD in order to simplify scanning procedures in this vulnerable population and determine minimum necessary scan time for these methods.

## 2. Materials and Methods

### 2.1. Participants

Three healthy volunteers and 5 patients with PD were recruited for this study. PD participants were nondemented, had moderate disease severity ratings, and were asked to withdraw from their medications overnight prior to the PET scan. All patients were on dopaminergic medications to treat PD symptoms at the time of scanning (carbidopa/levodopa *n* = 5, pramipexole *n* = 4, amantadine *n* = 1, and entacapone *n* = 1). Participant and scan details are presented in Table 1.

### 2.2. Image Acquisition

[^18^F]SynVesT-1 was synthesized as previously described [10], with minor modifications [16]. PET scans were acquired on a GE Discovery 5-ring PET/CT scanner. Following a CT scan for attenuation correction, dynamic emission data were collected in list mode for 120 minutes beginning 30 seconds prior to radiotracer injection. Participants were situated on the scanner bed with a thermoplastic face mask (Tru-Scan Imaging, Annapolis, USA) fitted to minimize movement. 186 ± 11 MBq [^18^F]SynVesT-1 was administered via bolus injection to the antecubital vein. Injection details are included in Table 1. Arterial blood samples were drawn throughout the scan from a catheter placed in the radial artery using an automated blood sampling system (ABSS PBS-101, Comecer S.p.A., Italy) for the first 22 minutes as well as samples drawn manually at intervals (-5, 2.5, 7, 12, 15, 20, 30, 45, 60, 90, and 110 min relative to emission scan start) for construction of the metabolite-corrected arterial input function. One participant was removed from the scanner at 90 minutes postinjection due to discomfort. T1-weighted MRI scans were collected for anatomical coregistration on a 3-T GE Discovery MR750 scanner.

### 2.3. PET Data Processing

List mode data were binned into 34 frames (6 × 30 s, 3 × 60 s, 2 × 120 s, and 22 × 300 s) and reconstructed using filtered back projection. Data processing was performed using the PNEURO and PFUS modules of PMOD (version 4.2, Zurich, Switzerland). Frames were realigned to a midpoint frame using rigid registration to correct for interframe motion. Attenuation- and motion-corrected dynamic PET image was then aligned to each subject's T1 anatomical MRI image. Subject MRI was normalized and resliced to the MNI template, and a probabilistic tissue segmentation map was generated. The Hammers atlas [17] was used to define 28 target grey matter regions of interest (ROIs) across the whole brain. The CS ROI was defined as described in [14] based on erosion of the CS mask included in the Automated Anatomical Labelling Atlas-3 [18] to minimize spill-in for SV2A PET. The final 29-ROI mask was transformed into subject PET space and refined based on individual tissue segmentation to include only voxels containing grey matter (target ROIs) or white matter (CS reference region). TACs were extracted from the dynamic image within each ROI for kinetic analysis.

### 2.4. Blood Analysis

Manual blood samples were used to determine radioactivity in whole blood and in plasma after centrifugation. Additional samples were drawn to measure metabolites using column-switching high performance liquid chromatography. Free fraction in plasma was measured using an ultrafiltration technique [19]. Arterial input functions were generated using in-house software [20]. Briefly, plasma-to-whole blood ratio was determined from manual samples and used to convert ABSS data to a plasma measure. Dispersion and delay correction was performed as previously described [21]; then, manual and corrected automatic sample values were merged into a single curve. A Hill function was fitted to parent fraction data. The final arterial input function for each scan consists of the product of the merged plasma curve and the smoothed parent fraction curve.

### 2.5. Kinetic Modeling

Analysis of ROI TACs was performed using the PKIN module of PMOD. [^18^F]SynVesT-1 BP_ND_ was determined in each ROI using either the 1TCM or the SRTM fitted to count-weighted regional TACs. Both methods used centrum semiovale (CS) as a reference region with very low specific binding of SV2A [12, 14]. Potential group differences in CS were first evaluated by comparing ROI volume (refined using subject-specific tissue segmentation), total radiotracer volume of distribution (*V*_T_), and efflux rate constant *k*_2_ values between PD and HCs. For 1TCM, *V*_T_ was extracted from each ROI and BP_ND_ was determined using CS *V*_T_ as an estimate of nondisplaceable volume of distribution, BP_ND_^ROI^ = *V*_T_^ROI^/*V*_T_^CS^ − 1. For noninvasive modeling, an SRTM2 approach was used [22] which fixes the reference region efflux rate constant *k*_2_′. Two approaches were compared: (1) fixing the reference region efflux rate constant *k*_2_′ to a population value (pop − *k*_2_′) based on group average *k*_2_ in CS from 1TCM fits or (2) a two-step procedure in which *k*_2_′ is a free parameter determined in a coupled fit to generate a single shared estimate across grey matter regions, then fixed to that value for subsequent fitting to generate BP_ND_ estimates (coupled − *k*_2_′). Direct estimation of SRTM and the Logan reference region graphical approach [23] using the pop − *k*_2_′ value described above were also evaluated for comparison. Based on the results of time stability analyses in the other seven participants (see Results), the participant with a 90-minute scan was included in kinetic modeling comparisons but was not included in calculation of pop − *k*_2_′. In all cases, absolute agreement and bias in BP_ND_ were assessed relative to 1TCM BP_ND_ values at the subject level in correlation and linear regression analyses and by computing percent bias across ROIs (%bias = [BP_ND_ − BP_ND_^1TCM^]/BP_ND_^1TCM^, such that negative values indicate underestimation).

Based on the results from ROI analyses, SRTM2 was applied to generate parametric images to assess suitability for voxel-level analyses. Resulting parametric images were visually inspected for image quality (e.g., absence of extreme values). Average BP_ND_ values were extracted from the parametric images using the same atlas used in ROI analyses and compared to BP_ND_ from direct ROI-level TAC fits to assess numerical agreement in correlation analyses.

### 2.6. Time Stability

To determine minimum scan time required for noninvasive analysis, 1TCM and SRTM2 were fitted as described above to scan data from the seven full-length scans, truncated to length (*t*_max_) of 40, 60, 80, 90, or 100 minutes. Model fits, parameter uncertainty, and bias in BP_ND_ estimates relative to the full 120 min analysis were assessed.

## 3. Results

### 3.1. CS Reference Region

**Figure 1:**
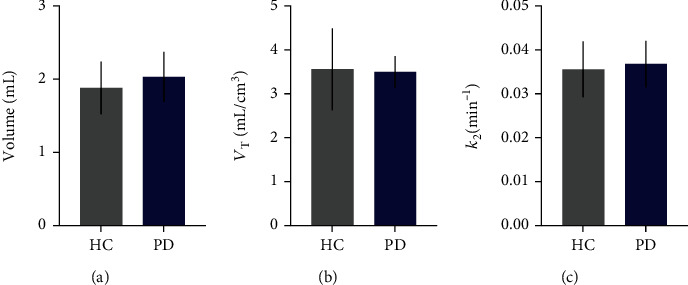
Centrum semiovale (CS) reference region in PD vs. HCs. No significant differences in (a) CS ROI volume, (b) radiotracer volume of distribution, or (c) efflux rate constant estimated by 1TCM in PD patients (*n* = 5) relative to HCs (*n* = 3).

The white matter CS region has low SV2A binding and can provide an estimate of nondisplaceable [^18^F]SynVesT-1 uptake for calculation of BP_ND_. Here, volume of the CS ROI did not differ between the HC and PD groups (HC 1.9 ± 0.4 cm^3^, PD 2.0 ± 0.3 cm^3^, *p* = 0.59). *V*_T_ in CS estimated using the 1TCM was also similar between groups (HC 3.6 ± 0.9 mL/cm^3^, PD 3.5 ± 0.4 mL/cm^3^, *p* = 0.93), as was the CS efflux rate constant *k*_2_ (HC 0.036 ± 0.006 min^−1^, PD 0.037 ± 0.005 min^−1^, *p* = 0.79). Altogether, these data support CS as a useful reference region in patients with PD, with no evidence of disease-related differences in target binding or efflux kinetics (Figure 1).

### 3.2. Noninvasive Modeling Approaches

**Figure 2:**
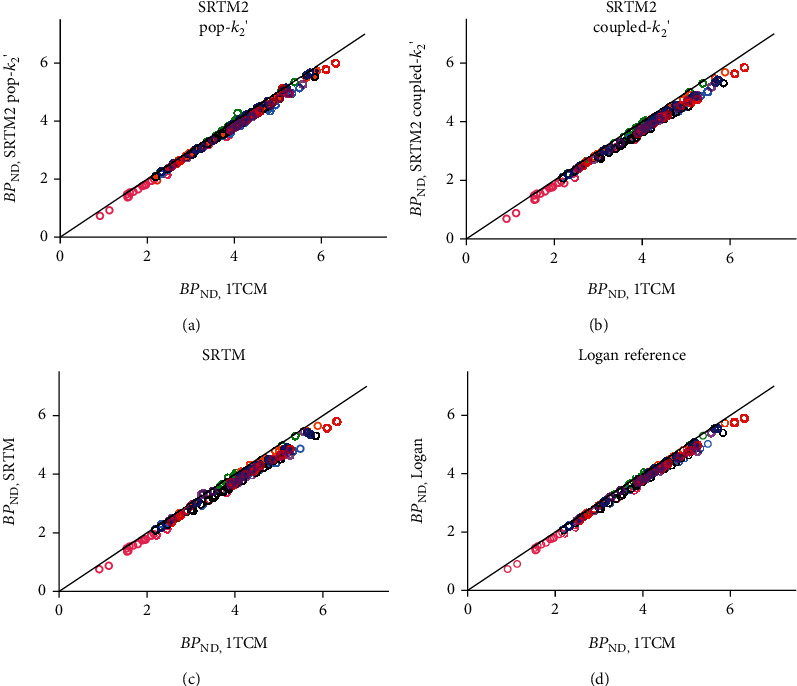
Agreement between BP_ND_ estimates derived from reference region quantification methods and those derived from 1TCM. Each method used CS as a reference region (see text). Points are ROI BP_ND_ values coloured according to the subject (*n* = 8).

**Figure 3:**
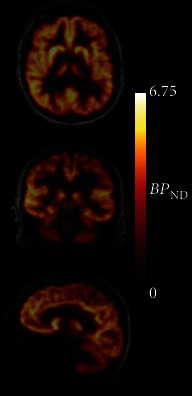
Parametric image of [^18^F]SynVesT-1 in a patient with PD. Voxel-level map of BP_ND_ generated using SRTM2 with pop − *k*_2_′ = 0.037.

Given the finding of no significant differences in CS parameters between HC and PD participants in this sample, a population estimate for the reference region efflux constant *k*_2_′ value was determined for subsequent analyses using the mean value across the full sample, pop − *k*_2_′ = 0.037 min^−1^. This is comparable to a previously reported pop − *k*_2_′ value for [^18^F]SynVesT-1 of 0.032 min^−1^ determined in 8 healthy control subjects [13]. SRTM2 model fits were good across grey matter ROIs on visual inspection, and parameter uncertainty was low (<10% relative standard error (rSE)). BP_ND_ values from SRTM2 using pop − *k*_2_′ = 0.037 min^−1^ showed excellent agreement with 1TCM values for every participant, with a small negative bias (*R*^2^s > 0.99; BP_ND_^SRTM2^ = 0.98^∗^BP_ND_^1TCM^ − 0.07; bias, HC −6.8% ± 4.6%, PD −4.8% ± 6.1%, *p* = 0.62) (Figure 2(a)). The coupled fitting method produced *k*_2_′ estimates slightly higher than pop − *k*_2_′ but again similar between groups (HC 0.042 ± 0.004 min^−1^, PD 0.042 ± 0.005 min^−1^, *p* = 0.89). BP_ND_ estimates using the coupled fit also showed good agreement with 1TCM results, low bias, and no evidence of group differences (*R*^2^s > 0.99; BP_ND_^SRTM2^ = 0.95^∗^BP_ND_^1TCM^ − 0.04; bias, HC −5.8% ± 3.1%, PD −6.3% ± 2.2%, *p* = 0.80), similar to pop − *k*_2_′ results (Figure 2(b)). Direct fitting of SRTM (i.e., without fixing *k*_2_′ across regions) produced similarly good agreement but slightly higher bias and intersubject variability compared to other methods, exceeding 15% bias in one participant (*R*^2^s > 0.98; BP_ND_^SRTM^ = 0.93^∗^BP_ND_^1TCM^ − 0.05; bias, HC −8.1% ± 7.4%, PD -6.2% ± 2.2%, *p* = 0.58) (Figure 2(c)). The Logan reference region fits were also tested using the same pop − *k*_2_′ value and produced similar results to coupled − *k*_2_′, with good agreement and low bias relative to 1TCM (*R*^2^s > 0.99; BP_ND_^Logan^ = 0.97^∗^BP_ND_^1TCM^ − 0.06; bias, HC −4.7% ± 3.7%, PD −5.1% ± 1.8%, *p* = 0.84) (Figure 2(d)). Linear relationships and bias across ROIs are presented for individual participants in Table 2. Altogether, in every case, using a reference region method produced values in very good agreement with those derived from the 1TCM, with bias around 5-8% across regions and no evidence of difference between patients and HCs. SRTM2 fitting methods were selected for further evaluation in parametric image and time stability analyses given their excellent agreement with 1TCM results across the full sample. Parametric BP_ND_ images were generated using SRTM2, which produced images of high quality with little visual evidence of excessive noise or extreme values.  Figure 3 shows a representative image of BP_ND_ in the brain of a PD patient generated using SRTM2 with pop − *k*_2_′ = 0.037 min^−1^. SRTM2 using either fixed pop − *k*_2_′ = 0.037 or the coupled *k*_2_′ method produced BP_ND_ values in good agreement with direct ROI modeling (subject *R*^2^s 0.93 ± 0.075, range 0.74–0.99), with a small negative bias (<10% in all ROIs).

### 3.3. Time Stability

**Figure 4:**
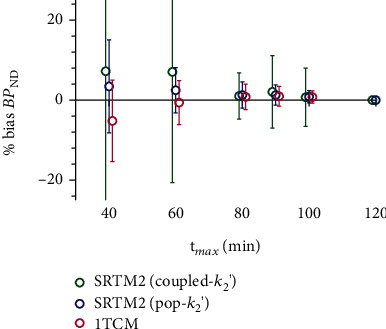
Bias in BP_ND_ estimates derived from SRTM2 or 1TCM. Values are presented relative to 120 min scan data fitted using the same method (*n* = 7).

For 1TCM with *t*_max_ = 60 min, absolute error in *V*_T_ vs. *V*_T(120 min)_ was <5% across most subjects and regions (*n* = 7; subject *R*^2^s > 0.99; bias, −0.6% ± 5.5%; bias in CS reference region, −0.5% ± 3.6%), consistent with a previous study at a different center [12]. With noninvasive methods, model fits were good and uncertainty in BP_ND_ estimates was low across the range of *t*_max_ values (rSE < 20%; with *t*_max_ ≥ 60 min, rSE < 10%). Using SRTM2 with pop − *k*_2_′ and *t*_max_ = 80 min, absolute error in BP_ND_ vs. BP_ND(120 min)_ was <5% (*R*^2^s > 0.97; bias, HC 1.2% ± 2.5%, PD 1.3% ± 2.5%, *p* = 0.95). Coupled − *k*_2_′ values from truncated scan data were higher than those derived using full data length (coupled − *k*_2__(80 min)_′ = 0.042 ± 0.004 min^−1^ in HCs and 0.039 ± 0.007 min^−1^ in PD). Despite this, bias in BP_ND_ using the two-step coupled − *k*_2_′ SRTM2 method was low and comparable between groups (*R*^2^s > 0.99; bias, HC −0.33% ± 4.5%, PD 2.0% ± 4.1%, *p* = 0.35) with *t*_max_ = 80 min. With *t*_max_ = 60 min, slightly higher bias and variability across subjects were observed (average bias 7.1% ± 28%), with one PD patient showing markedly (~50%) higher BP_ND_ estimates.  Figure 4 shows bias in BP_ND_ estimates at each tested *t*_max_ value relative to *t*_max_ = 120 min, showing that accurate quantification could be achieved in both groups with 60 min scan time using arterial blood sampling and the 1TCM and with 80 min scan time using SRTM2.

## 4. Discussion

Simplified quantification methods for synaptic density PET with [^18^F]SynVesT-1 would greatly facilitate its widespread use as a radiopharmaceutical for clinical PET research in PD. Here, noninvasive modeling techniques produced excellent results that agreed closely with parameter estimates derived using full arterial sampling. SRTM2 using either a pop − *k*_2_′ value (0.037 min^−1^) or a two-step coupled fitting method produced BP_ND_ estimates with low bias and, critically, no evidence of group differences in agreement or variability. Bias was under 5% in most cases and under 10% for virtually all regions and participants. Scan time as short as 80 minutes provided sufficient data to obtain stable parameter estimates in good agreement with those derived from 120 min data using these methods.

The CS has previously been investigated as a reference region for SV2A PET [14, 15]. Here, there was no evidence of differences in reference region *V*_T_ or efflux between healthy people and individuals with PD, suggesting that differences in central white matter integrity are unlikely to interfere with synaptic density quantification in this sample. Nevertheless, it may be the case that in individual subjects, changes in white matter tissue characteristics through degradation or other disease processes could potentially influence binding parameters or data quality in this small region. This possibility should be considered as a point of caution for future studies in larger and more heterogeneous samples. Studies of PD and other neurodegenerative disorders may benefit from evaluating white matter volume differences to assess the likelihood of this potential confound.

All reference region quantification methods produced good fits to the data and BP_ND_ values in close agreement with those derived from 1TCM. SRTM2 with pop − *k*_2_′ performed best, though these results should be interpreted with caution given the low sample size for analyses and for determination of the pop − *k*_2_′ value. SRTM2 with coupled − *k*_2_′ estimates produced similarly excellent results, with bias < 10% in all cases. This is a particularly attractive method given that it does not rely on prior determination (using arterial blood sampling) of the pop − *k*_2_′ value and thus is fully noninvasive and not dependent on assumptions about group homogeneity. The Logan reference region graphical method (*k*_2_′ = 0.037 min^−1^, *t*^∗^ = 30 min) and direct fitting of SRTM also produced BP_ND_ values that were generally in good agreement with the 1TCM, but SRTM results were more variable across participants. Given the low sample size, it cannot be determined with confidence in the present sample that this method would be broadly reliable across participants, and so we consider SRTM2 or the Logan reference region methods preferable at this stage. Nevertheless, results for all methods, and for SRTM2 in particular given its analytical complexity, remain preliminary given the low sample size. These methods require further evaluation in larger, more diverse, and external samples.

CS *k*_2_ estimates from 1TCM in this sample showed good agreement between PD and HCs. Given negligible differences between the groups, a mean value across all participants was selected as the pop − *k*_2_′ for SRTM2 analyses. This value is similar to, but slightly higher than, a value previously reported for HCs from another center (0.037 min^−1^ vs. 0.032 min^−1^) [13]. Inclusion of PD patients may contribute to some of this difference, though values from HCs alone were also higher (0.036 min^−1^, *n* = 3). The higher CS *k*_2_ values in the present work may reflect technical differences, including scanner type (GE Discovery MI 5-ring PET/CT vs. HRRT) which have different image resolutions, and arterial sampling methods (automatic vs. rapid manual sampling). To explore effects of these different values, analyses were repeated using SRTM2 with the previously reported pop − *k*_2_′ value of 0.032 min^−1^, which also produced excellent results (BP_ND_*R*^2^s > 0.99; mean bias −1.6% ± 1.5%). As in the previous work [13], *k*_2_′ estimates from coupled fits in individual subjects were consistently higher than the pop − *k*_2_′ derived from modeling with blood data. However, bias was comparable between the two SRTM2 methods. Given modest sample size in these analyses, these results should be considered preliminary, but the excellent agreement and consistency of these results at the individual level across the full sample give confidence that SRTM2 quantification will produce reliable results.

At the voxel level, SRTM2 fitted using either the pop − *k*_2_′ or the coupled fitting method produced high-quality parametric images of BP_ND_ in HC and PD groups. Average ROI BP_ND_ values extracted from parametric images correlated closely with those from direct fits to ROI TACs. Though a small (<10%) negative bias in BP_ND_ was observed, this was consistent across groups. Therefore, parametric image analysis may be possible to evaluate spatially restricted or variable patterns of difference in synaptic density or in other whole brain analyses, such as multimodal imaging comparisons.

Time stability analysis replicated a previous finding that scan time as short as 60 minutes may be sufficient with arterial sampling [12], demonstrating consistency in a different center and scanner type. Minimum scan duration was also assessed using SRTM2 in order to develop a patient-optimized protocol that would minimize discomfort without sacrificing quantitative accuracy. Using SRTM2, scan duration of 80 minutes or more produced results in excellent agreement with 120 min scans, suggesting that the full two-hour scan time is not necessary for accurate quantification in this study population. We noted progressively greater error in *V*_T_ estimates with scan times shorter than 60 minutes, indicating that information about radiotracer washout from brain within 30-60 minutes postinjection is necessary to model the system accurately. Notably, in this study, one participant in the PD group was not able to complete the full 120 min scan time and was removed from the scanner at 90 minutes due to discomfort, underlining the importance of determining the minimum necessary scan time. Results from the remaining seven participants suggest that this 90 min scan was still sufficient for accurate quantification. Shorter scan times would improve patient comfort and may avoid such cases. This is a key consideration in elderly patient populations, which also reduces the risk of motion artefacts and technical issues as scans progress. This revised acquisition strategy which decreases scanning time and avoids arterial blood measurements would improve patient recruitment and throughput, particularly from a single synthesis of this fluorine-18 labelled radiopharmaceutical, thereby facilitating multicenter clinical trials. These methods will also reduce costs and increase the clinical utility of [^18^F]SynVesT-1 PET.

## 5. Conclusions

Preliminary analyses in a small sample of participants found that noninvasive quantification produced BP_ND_ values in excellent agreement with 1TCM values for HCs and patients with PD. This requires confirmation in larger samples, and it remains to be determined to what extent these results can be extrapolated to other samples, including PD patients that differ in key characteristics like age or disease severity. Nevertheless, these results suggest that accurate [^18^F]SynVesT-1 quantification could be performed noninvasively in individuals with PD and with scan durations as short as 80 minutes. Each of these factors will greatly improve patient comfort and throughput in studies of PD and facilitate the widespread use of [^18^F]SynVesT-1 as a biomarker of brain integrity in neurological disorders.

## Figures and Tables

**Table 1:** Participant and scan characteristics.

	Healthy control	Parkinson's disease	*p*
*N* (*n* male)	3 (2)	5 (4)	
Age (years)	54 ± 11	66 ± 7	0.11
Disease duration (years)	—	11.4 ± 4.5	—
LEDD (mg)	—	1,127.8 ± 630.9	—
MDS-UPDRSIII score	—	34.8 ± 12.8	—
Hoehn and Yahr scale score	—	2.6 ± 0.4	—
Injected activity (MBq)	185 ± 2.0	186 ± 13.1	0.91
Molar activity (MBq/nmol)	100 ± 53	175 ± 130	0.39
Plasma free fraction	26.1 ± 2.6	28.6 ± 2.8	0.34

*p* values from independent samples *t*-tests comparing HC to PD. Values represent group mean ± SD. Abbreviations: LEDD: L-Dopa equivalent daily dose; MDS-UPDRSIII: Movement Disorders Society-Unified Parkinson's Disease Rating Scale, part III motor assessment.

**Table 2:** Subject-level relationship between BP_ND_ estimates from noninvasive methods and BP_ND_ estimates from 1TCM.

Method	HC1	HC2	HC3	PD1	PD2	PD3	PD4	PD5
SRTM2, *pop* − *k*_2_′								
*Equation*	0.93*x* − 0.035	1.02*x* − 0.13	0.94*x* + 0.019	0.98*x* − 0.013	0.92*x* + 0.071	1.00*x* − 0.090	0.95*x* + 0.015	0.94*x* + 0.030
*Bias*	−8.1% ± 4.5%	−1.9% ± 1.6%	−5.2% ± 0.7%	−2.7% ± 0.1%	−6.5% ± 1.1%	−2.0% ± 0.5%	−4.7% ± 1.0%	−5.2% ± 0.3%
SRTM2, *coupled* − *k*_2_′								
*Equation*	0.92*x* − 0.044	1.00*x* − 0.096	0.88*x* + 0.12	0.97*x* + 0.0094	0.90*x* + 0.13	0.93*x* + 0.098	0.90*x* + 0.13	0.92*x* + 0.078
*Bias*	−9.5% ± 5.7%	−2.6% ± 0.9%	−8.6% ± 1.4%	−3.3% ± 0.1%	−7.1% ± 1.2%	−4.8% ± 0.6%	−6.9% ± 1.0%	−6.5% ± 0.7%
SRTM								
*Equation*	0.84*x* + 0.14	1.00*x* − 0.10	0.88*x* + 0.11	0.95*x* + 0.071	0.87*x* + 0.25	0.93*x* + 0.095	0.89*x* + 0.16	0.87*x* + 0.29
*Bias*	−16.6% ± 23.8%	−2.3% ± 1.8%	−8.7% ± 1.3%	−3.1% ± 1.2%	−7.0% ± 2.6%	−4.7% ± 1.1%	−6.8% ± 1.6%	−5.7% ± 3.0%
Logan reference								
*Equation*	0.92*x* − 0.023	1.00*x* + 0.096	0.92*x* + 0.049	0.98*x* + 0.0013	0.90*x* + 0.14	0.98*x* − 0.048	0.93*x* + 0.087	0.92*x* + 0.14
*Bias*	−8.9% ± 3.7%	−2.4% ± 1.4%	−7.1% ± 0.8%	−2.3% ± 0.7%	−6.2% ± 1.5%	−2.9% ± 0.9%	−5.0% ± 0.8%	−4.7% ± 1.5%

*Equation* is the fitted linear relationship between BP_ND_ values from the specified method and those from 1TCM. *Bias* is mean ± s.d. percent difference in BP_ND_ estimates across ROIs. BP_ND_ estimates for PD5 (all methods) were derived from a 90 min scan. See text for details of quantification methods.

## Data Availability

Data is available upon request to the corresponding author.
